# Supplementary material for: Complexome profiling of the Chlamydomonas psb28 mutant reveals TEF5 as an early PSII assembly factor
Source: Plant Cell. 2025 Mar 17;37(6):koaf055. doi: 10.1093/plcell/koaf055 (PMC12164590; doi:10.1093/plcell/koaf055)
Supplement: koaf055_Supplementary_Data [file koaf055_supplementary_data.zip › psb28_interactive.zip!/plots/intensity/qualityPlots/peptides/PTOX2_Cre03.g172500.t1.1.html]

### Cre03.g172500.t1.1

Trivial name: PTOX2  
SeqCoverage [median]: 29.10  
Mass: 46.77 kDa  
  
 

| Sequence | MissedCleavage | Mass [Da] | wtIntensity | muIntensity | Score |
| --- | --- | --- | --- | --- | --- |
| AFGSLDK | 0 | 736.38 | 0.00 | 2504.38 | 72.14 |
| AKLDKLELSVSDVVAVEHAR | 2 | 2178.20 | 0.00 | 3676.70 | 32.16 |
| DIAASSSGSAPR | 0 | 1117.54 | 4858.75 | 12019.27 | 130.41 |
| DNYLHEFTQLR | 0 | 1434.69 | 0.00 | 3521.29 | 67.15 |
| DNYLHEFTQLRR | 1 | 1590.79 | 714.19 | 3310.04 | 37.18 |
| LYQEGYGEVPK | 0 | 1281.62 | 0.00 | 328.00 | 56.43 |
| LYQEGYGEVPKDVWQLAK | 1 | 2122.07 | 0.00 | 969.02 | 32.69 |
| MAYNFMQR | 0 | 1059.46 | 0.00 | 3781.81 | 102.98 |
| STRYPPSDITAPDGAVAK | 1 | 1844.93 | 0.00 | 591.22 | 55.33 |
| VELHAADTYSAFVER | 0 | 1706.83 | 1595.93 | 9433.75 | 129.38 |
| VLHSAGAAAAR | 0 | 1022.56 | 0.00 | 1847.30 | 90.66 |
|  |  |  | 7168.9 | 41982.8 |  |
